# Case of Persistent Facial Neuralgia of Reflex Dental Origin Due to an Unerupted Cuspid Tooth
*Reported before the First District Dental Society of the State of New York February 12, 1889; (Dental Cosmos).


**Published:** 1889-12

**Authors:** Edward C. Kirk

**Affiliations:** Lecturer on Operative Dentistry, University of Pennsylvania.


ARTICLE III.

### CASE OF PERSISTENT FACIAL NEURALGIA OF REFLEX DENTAL ORIGIN DUE TO AN UNERUPTED CUSPID TOOTH.*

BY EDWARD C. KIRK, D. D. S.

[Lecturer on Operative Dentistry, University of Pennsylvania.]

I have to report a case which has lately been under my care, and which has been quite interesting to me. A lady was referred to me by Dr. George T. Stevens, of New York City, with a request that I would examine her mouth, to ascertain if any dental origin could be assigned for a severe and persistent facial neuralgia from which she had suffered for between three and four years, with but slight periods of intermission. She was markedly neurasthenic, and had been under Dr. Stevens' care for a complicated ocular defect, caused by a lack of parallelism in the visual axes, due to an insufficiency of the recti muscles. The continued effort to accomplish binocular vision under these conditions had resulted in an amount of nervous strain which, among other complications, resulted in pronounced reflex gastric disturbance in the form of nausea, indigestion, etc., with a general condition of intense nervous irritability. The eye phase of the trouble had been almost entirely corrected by Dr. Stevens, and a general improvement in her gastric difficulty had occurred. The facial neuralgia was persistent, and, suspecting that it might be of reflex dental origin, he asked me to examine the case from that standpoint.

The upper jaw I found to be edentulous, the loss of the natural teeth being supplied by an entire artificial denture, the lower teeth back of the cuspids were also

---

*** Reported before the First District Dental Society of the State of New York February 12, 1889; (Dental Cosmos).**

missing. Those that remained were in a perfectly healthy condition, and a careful examination of the mouth and jaws elicited nothing abnormal. An examination of the nasal chamber showed slight congestion and hypertrophy of the mucous membrane on the left side, and there was a history of occasional discharges of bloody mucus from the nostril. She stated also that at the time of the extraction of the wisdom tooth on the upper left side her dentist had discovered at the bottom of the socket a portion of necrosed bone, for which he, in connection with her family physician, treated her for some months by local medicinal applications. The socket finally healed. While the symptoms were not typical of catarrhal antral disease, I considered there were sufficient indications of antral trouble to warrant me in opening that cavity, which I did by perforating the alveolar ridge at the position of the second bicuspid tooth. An exploration of the antrum revealed a spot of necrosed bone in the floor of the cavity, at about the position of the second molar tooth, and also a necrosed condition of the edges of the antral foramen connecting that cavity with the nose. The dead bone was removed with burs and spoon excavators, and the surface treated with applications of aromatic sulphuric acid until the process of healing was completed, which occurred between three and four weeks afterwards. During this time, and subsequently, there was no abatement of the neuralgic difficulty. As I could discover nothing further. I referred her to Dr. Stevens again for a final correction of her eye difficulty, telling her that after this was done, and if she found no improvements in her neuralgia, to come back to me, and I would again go over her case to see if I could discover anything further which had any bearing upon it. Nearly three months afterwards she returned, stating that her pain continued as before, and that her life was becoming an intolerable burden by reason of it. The idea of an unerupted tooth occurred to me, and I casually asked the question if she could remember if she had erupted all her teeth, or if any of them were missing from the arch. She said that she was "quite positive" that she had "never had an eye-tooth on the left side." As she had not the slightest doubt in regard to this fact, I at once perforated the outer alveolar plate at the position of the canine eminence, and, on passing a probe, discovered a cavity in the bone which was abnormal. I was, however, unable to detect the presence of a tooth. I proposed to her a more extended operation for the purpose of deciding as to the existence of an unerupted tooth, to which she promptly acceded. On the following Sunday, in the presence of Drs. Bonwill, C. H. Thomas, and Schneideman, I removed a portion of the outer alveolar plate, about half an inch long, between the position of the left central incisor and cuspid teeth, and found an unerupted cuspid lying in a horizontal position, with the apex of the root in the forward end of the antrum, and the point of the crown imbedded in the symphysis of the superior maxillæ immediately below the floor of the nose, the whole tooth occupying a position at least half an inch back from the anterior alveolar plate. The extraction of the tooth was effected by making a section of it at about the middle of its length by means of a large fissure bur, after which the root end was first extracted with forceps, followed by the removal of the crown in the same manner.

The after-treatment of the case consisted in washing out the cavity and antrum with diluted phenol sodique through the wound, which was partially closed with sutures at each side, the central portion being allowed to remain open to secure drainage. I have here the specimen, which is presented for your inspection. The effect upon the neuralgia has been noticeable, although the operation is of too recent date (two weeks ago this Sunday) to enable me to report definitely as to the permanence of the good so far obtained.† She has had several attacks of pain, but they

---

† Since the report was made, now over a month ago, the facial neuralgia has entirely disappeared, and the healing process is so far completed that the external wound will barely are not so persistent, nor are they of the same character as that from which she previously suffered ; and it is my belief that when the process of healing is complete, her neuralgia, at least from this source, will disappear.—*Univ. Med. Magazine.*

admit a silver probe the thickness of a small knitting needle. A slight attack of acute coryza during the period of healing caused some hyperactivity of the antral mucous membrane, which yielded promptly to injections of an aqueous solution of resorcin, ten grains to the ounce. A noticeable improvement in the general condition of the patient has taken place particularly as to nervous tone, bodily weight, spirits, etc. Her appetite is improved, she is able to secure uninterrupted sleep at night, and expresses herself as feeling entirely well.

